# Serological Detection of Antibodies Against *Mycoplasma bovis* Infection by Competitive Enzyme-Linked Immunosorbent Assay (cELISA)

**DOI:** 10.3390/vetsci13080741

**Published:** 2026-07-25

**Authors:** Wentao Fei, Li Yang, Yuhao Zhao, Zhijie Xiang, Chengwei Fang, Yingyu Chen, Changmin Hu, Aizhen Guo

**Affiliations:** 1 National Key Laboratory of Agricultural Microbiology, College of Veterinary Medicine, Huazhong Agricultural University, Wuhan 430070, China; feiwentao@163.com (W.F.); zhaoyuhao@webmail.hzau.edu.cn (Y.Z.); xiangzhijie2024@163.com (Z.X.); fangchengwei@webmail.hzau.edu.cn (C.F.); chenyingyu@mail.hzau.edu.cn (Y.C.); hcm@mail.hzau.edu.cn (C.H.); 2 Key Laboratory of Development of Veterinary Diagnostic Products, Ministry of Agriculture and Rural Affairs, Huazhong Agricultural University, Wuhan 430070, China; 3 Hubei Hongshan Laboratory, Huazhong Agricultural University, Wuhan 430070, China; 4 Wuhan Keqian Biology Co., Ltd., Wuhan 430206, China; yangli15527183902@163.com

**Keywords:** *Mycoplasma bovis*, competitive ELISA, MbovP579, serodiagnosis, bovine respiratory disease

## Abstract

*Mycoplasma bovis* (*M. bovis*) is a major pathogen in cattle, primarily responsible for bovine respiratory disease complex (BRDC). Characterized by high infectivity and morbidity, *M. bovis* spreads rapidly within herds and is challenging to control and eradicate, underscoring the need for early and accurate diagnosis. To address this, we developed a competitive enzyme-linked immunosorbent assay (cELISA) for detecting *M. bovis*-specific antibodies in bovine sera. The assay uses recombinant MbovP579 protein (1 μg/mL) as the coating antigen and an HRP-conjugated 1A2 monoclonal antibody against MbovP579 (mAb, 0.35 μg/mL) as the competitor. With a cut-off value of 40.69% (percentage inhibition, PI), the cELISA demonstrated apparent sensitivity (90%; 95% CI: 78.64–95.65%) and specificity (96.15%; 95% CI: 90.53–98.49%) using the known background positive and negative sera. In addition, it yielded 100% positivity (9/9) against sera collected from 21 days post-immunization. The assay showed no cross-reactivity with other *Mycoplasma* species or two common bovine bacterial pathogens, confirming its high specificity. The intra- and inter-assay coefficients of variation (CVs) were below 10%. In clinical evaluations, this cELISA generated a 55.57% positivity rate for field samples (*n* = 1069) and exhibited 90.46% overall agreement (967/1069; 95% CI: 88.54–92.15%) with the commercial indirect ELISA kit, demonstrating strong diagnostic consistency. Discrepant specimens were further analyzed by a metabolic inhibition test (MIT), which suggested improved specificity, although further validation is required of this cELISA.

## Simple Summary

*Mycoplasma bovis* (*M. bovis*) is a highly infectious pathogen associated with bovine respiratory disease complex (BRDC) and high morbidity in cattle, highlighting the need for early and accurate diagnosis. In this study, a competitive enzyme-linked immunosorbent assay (cELISA) was developed to detect *M. bovis*-specific antibodies in bovine serum. The assay used recombinant MbovP579 protein (1 μg/mL) as the coating antigen and an HRP-conjugated anti-MbovP579 monoclonal antibody 1A2 (0.35 μg/mL) as the competing reagent. The cut-off value was established at 40.69% percentage inhibition. Validation with known positive and negative sera yielded an apparent sensitivity of 90.00% (95% CI: 78.64–95.65%) and a specificity of 96.15% (95% CI: 90.53–98.49%). The assay detected antibodies in all sera collected 21 days after immunization (9/9), showed no cross-reactivity with other *Mycoplasma species* or main common bovine bacterial pathogens, and had intra- and inter-assay coefficients of variation below 10%. Among 1069 field samples, 55.57% tested positive. Agreement with a commercial indirect ELISA was 90.46% (95% CI: 88.54–92.15%). Metabolic inhibition testing of discrepant samples indicated enhanced specificity, supporting the cELISA as a reliable tool for *M. bovis* serodiagnosis.

## 1. Introduction

*Mycoplasma bovis* (*M. bovis*) is a significant pathogen responsible for bovine respiratory disease complex (BRDC) and systemic infections in cattle, including pneumonia, mastitis, arthritis, otitis, keratoconjunctivitis, and reproductive disorders [1,2]. Neonatal and weaned calves, as well as cattle subjected to environmental stressors (e.g., transportation, regrouping, or seasonal changes), are particularly susceptible. The absence of a cell wall renders *M. bovis* intrinsically resistant to β-lactam antibiotics (e.g., penicillin) and prone to developing resistance to other antimicrobials [3,4]. Compounded by the lack of effective vaccines and targeted therapeutics, *M. bovis* has emerged as a critical threat to global cattle production, with severe economic consequences for both dairy and beef industries [3,5,6].

Systematic analyses reveal that *M. bovis* infections lead to substantial economic losses, including a 15–30% decline in milk production, elevated somatic cell counts (>500,000 cells/mL) [7,8], and increased mortality and culling rates (5–20%) [9,10,11]. Chronic infections and synergistic interactions with respiratory pathogens (e.g., *Mannheimia haemolytica*) further exacerbate disease severity and complicate treatment [12]. These challenges highlight the urgent need for reliable diagnostic and control strategies, particularly given the pathogen’s rapid transmission within herds and persistence once established [13].

Current diagnostic methods for *M. bovis* include pathogen isolation, molecular techniques (e.g., PCR), and serological assays [14,15]. However, chronic infections and prolonged antibiotic use often hinder bacterial culture, while serological tests, especially enzyme-linked immunosorbent assays (ELISAs), offer high sensitivity and specificity for detecting antibodies in infected cattle [16,17]. Among these, the competitive ELISA (cELISA) exhibits prominent advantages: it quantifies target antibodies via competitive binding against specific epitopes, which effectively reduces cross-reactivity induced by contaminating proteins present in coated antigens [9,18]. Accordingly, cELISA may improve specificity when based on well-characterized antigen–antibody competition systems [19].

In our previous work, using a proteomics approach, we identified the *M. bovis* membrane protein MbovP579 and demonstrated that it is a conserved, sensitive, and specific antigen among *M. bovis* strains, indicating its potential as a diagnostic target [10]. Subsequently, a monoclonal antibody was developed [20]. Here, we developed a cELISA using recombinant MbovP579 as the coating antigen and a monoclonal antibody (1A2) as the competitive probe. This assay aims to provide a cost-effective, high-throughput tool for accurate *M. bovis* serodiagnosis, supporting improved disease management in clinical and field settings.

## 2. Materials and Methods

### 2.1. Animal Ethics

Ten-week-old BALB/c mice were obtained from the Experimental Animals of Huazhong Agricultural University and used to develop the mAbs. Naturally immunized cattle and *M. bovis*-negative cattle from a farm in Hubei Province, China, were sampled with the consent of the farm owners. The animal experiments in this study were conducted in strict accordance with the Guide for the Care and Use of Laboratory Animals, Hubei Province, China. The protocols were approved by the Ethics Committee of Huazhong Agricultural University (Agreement No. HZAUMO-2023-0038; Agreement No. HZAUCA-2022-0017).

### 2.2. Serum Sample Collection

The negative serum samples for *M. bovis* in each experiment were verified using a commercial *M. bovis* antibody test kit (Biovet^®^, Saint-Hyacinthe, QC, Canada) and PCR [11,21], corresponding to cattle that showed no clinical symptoms [22,23]. Information regarding the positive serum samples for experimental groups is presented in Table 1.

Antisera against the *M. bovis* HB150 strain, *Mycoplasma bovirhinis*, *Mycoplasma agalactiae*, *Mycoplasma arginini*, *Pasteurella multocida*, and *Mannheimia haemolytica* were prepared via vaccination. For *M. bovis*, calves were immunized with an attenuated *M. bovis* HB150 strain (antigen concentration of 10^8^ CFU/mL). Negative calves were intranasally inoculated with 2 × 10^8^ CFU/2.0 mL. Blood samples were obtained from the caudal (tail) or jugular vein at 0, 7, 14, 21, 28, and 35 days post-vaccination, and positivity was determined using the Biovet^®^ kit.

All the serum samples were subsequently stored at −20 °C in the National Key Laboratory of Agricultural Microbiology, Huazhong Agricultural University.

For the other pathogens, aluminum hydroxide gel-inactivated vaccines were prepared using *Mycoplasma bovirhinis*, *Mycoplasma agalactiae*, *Mycoplasma arginini*, *Pasteurella multocida*, and *Mannheimia haemolytica* via a 60 °C water bath treatment for 30 min (antigen concentration of 10^9^ CFU/mL). Negative calves were subcutaneously inoculated with 2 × 10^9^ CFU/2.0 mL and boosted with the same dose 30 days later. Blood samples were collected one week post-boost, and positive sera were confirmed via an in-house indirect ELISA.

For the in-house iELISA, ultrasound-fragmented bacterial proteins (quantified by an Enhanced Bicinchoninic Acid Protein Assay kit) were coated at 1.0 μg/mL overnight at 4 °C, blocked with 200 μL 1% fish gelatin (Sigma, Burlington, MA, USA) in PBS for 1 h at 37 °C, and incubated with serum samples. Following standard washing with PBST, the plates were incubated with TMB (Seracare, Shanghai, China) for 10 min in the dark, stopped with 2 M H_2_SO_4_, and read at 450 nm using a microplate reader (BMG LABTECH, Offenburg, BW, Germany). Samples were classified as positive if their OD_450_ value was ≥2.1 times that of the negative control.

### 2.3. Expression and Purification of MbovP579 Protein

The recombinant plasmid encoding the MbovP579 protein was previously constructed in our lab. Briefly, the *Mbov579* DNA fragment was inserted into the pET30a(+) plasmid and transformed into *E. coli* BL21 (DE3) for expression [10]. MbovP579 was purified by a Ni Sepharose^TM^ 6 Fast Flow resin gravity column (GE Healthcare, IL, USA). Purity was evaluated by 10% sodium dodecyl sulfate–polyacrylamide gel electrophoresis (SDS-PAGE), and the concentration was determined by the Enhanced BCA Protein Assay kit (Beyotime, Shanghai, China).

### 2.4. Production of Monoclonal Antibodies Against the MbovP579 Protein

Three hybridoma cell lines, 1A2, 4C9, and 4C11, were developed with the procedure as follows. Briefly, BALB/c mice were immunized with purified MbovP579 antigen emulsified in Freund’s adjuvant. Spleen cells were fused with SP2/0 myeloma cells using PEG-mediated fusion, and positive clones were selected using in-house iELISA. Furthermore, 10-week-old SPF BALB/c mice were injected with 500 µL of Freund’s incomplete adjuvant (IFA, Sigma, USA), followed 5–7 days later by intraperitoneal injection of hybridoma cells (5 × 10^5^ to 1 × 10^6^) to generate ascites. Ascites fluid was collected and purified using rProtein G Beads 4FF columns (Smart Life Sciences, Nanjing, China), and the purified monoclonal antibodies were quantified using the BCA assay.

### 2.5. HRP-Conjugated mAbs and Blocking Assay Evaluation

Monoclonal antibody labeling was performed using a modified periodate oxidation method. Briefly, 5 mg of HRP (Sigma, USA) was dissolved in 1 mL of double-distilled water, mixed with 500 µL of 0.1 M sodium periodate solution, and incubated at 4 °C for 30 min. Then, 0.5 mL of 0.16 M ethylene glycol was added, reacting at room temperature in the dark for 30 min. Purified mAb (5 mg) was added, and the solution was dialyzed against 0.05 M carbonate–bicarbonate buffer (pH 9.5) at 4 °C for 15 h. Subsequently, 0.2 mL of 4 mg/mL NaBH_4_ (Sinopharm, Beijing, China) was added and incubated at 4 °C for 2 h. The conjugate was precipitated with saturated ammonium sulfate, centrifuged, resuspended in phosphate buffer (PB), and dialyzed. The HRP-conjugated mAbs were verified via iELISA and stored at −80 °C.

To validate blocking efficacy, a cELISA was performed. Plates were coated with MbovP579 protein (100 ng/well, 100 µL/well) at 4 °C for 12 h, blocked with 5% skim milk (200 µL/well) at 37 °C for 60 min, and washed. Negative and positive sera (100 µL/well) were incubated at 37 °C for 60 min. After washing, HRP-labeled mAbs (1A2, 4C9, 4C11) at dilutions of 1:1000, 1:2000, and 1:5000 were added and OD_450_ was measured. The percentage inhibition (PI) was calculated as follows [24,25]:

$$\mathrm{PI}\;(\%)=\left(1-\frac{{\mathrm{OD}}_{\mathrm{Sample}}}{{\mathrm{OD}}_{\mathrm{Negative}\;\mathrm{Control}}}\right)\times 100\%$$

### 2.6. Establishment of MbovP579-cELISA

The optimal coating concentration of MbovP579 (0.5–4.0 μg/mL) and dilution of HRP-conjugated 1A2 mAb (0.28–2.8 μg/mL) were determined by checkerboard titration. The ratio of OD_450_ values of the negative control serum (N) to the positive control serum (P), expressed as N/P, was calculated. The conditions yielding the highest N/P ratio were considered optimal. Optimal serum dilution (1:2–1:10) and reaction times (45, 60, or 90 min) were subsequently determined using the same maximization principle.

### 2.7. Determination of Cut-Off Value, Diagnostic Sensitivity, and Specificity

Fifty *M. bovis*-positive (Table 1) and 104 *M. bovis*-negative (Table A3) serum samples were tested to determine the cut-off value. The diagnostic cut-off was established by receiver operating characteristic (ROC) curve analysis. The optimal threshold was selected to maximize Youden’s index (J = sensitivity + specificity − 1). Then, diagnostic sensitivity and specificity were calculated based on a 2 × 2 contingency table of true disease status vs. test results. Diagnostic sensitivity was defined as the ability of the assay to correctly identify *M. bovis*-positive samples, whereas diagnostic specificity was defined as the ability of the assay to correctly identify *M. bovis*-negative samples [26].

### 2.8. Evaluation of cELISA

Sensitivity was evaluated using two approaches. First, nine clinically healthy calves confirmed to be negative for *M. bovis* were inoculated intranasally with the *M. bovis* HB150 strain at a dose of 2 × 10^8^ CFU in 2.0 mL. Blood samples collected at 0, 7, 14, 21, 28, and 35 days post-inoculation were tested in parallel using the cELISA and the Biovet^®^ kit. Second, strongly positive serum samples were subjected to serial dilutions ranging from 1- to 64-fold to determine the analytical limit of detection, and the results were compared with those obtained using the commercial Biovet^®^ kit.

Analytical specificity was assessed by testing antisera against *M. bovis* HB150, *M. bovirhinis*, *M. agalactiae*, *M. arginini*, *P. multocida* and *M. haemolytica*.

Repeatability was assessed by testing 10 positive and 10 negative samples in triplicate across three plates from the same batch (intra-assay) and different batches (inter-assay). The coefficient of variation (CV) was calculated using the formula CV = SD/X × 100%, where SD represents the standard deviation, and X represents the mean PI value [27].

### 2.9. Metabolic Inhibition Test (MIT) of M. bovis

*M. bovis* cultured to the logarithmic phase was diluted to 10^4^ CFU/mL using PPLO liquid medium for use. For the assay, eight wells were designated on a cell culture plate, including 3 test wells, 3 negative serum control wells, and 2 blank control wells.

Aliquots of the diluted bacterial suspension (100 μL per well) were dispensed into both test wells and negative serum control wells. Subsequently, 100 μL of *M. bovis* antiserum was pipetted into the test wells, while 100 μL of antibody negative serum was added to the negative serum control wells. All mixtures were gently mixed to ensure homogeneity. Finally, 200 μL of PPLO medium alone was added to the blank control wells.

The plate was incubated at 37 °C for 48 h prior to result observation. The anticipated outcomes are as follows: no noticeable color change should be observed in the medium of test wells and blank control wells, whereas the medium in negative serum control wells should turn distinctly yellow [28].

### 2.10. Application for the cELISA

The established cELISA was used to test 1069 clinical bovine serum samples from four different farms. The results were compared with the commercial Biovet^®^ kit to calculate the sensitivity and agreement. Discrepant specimens were further characterized by the metabolic inhibition test (MIT) to facilitate subsequent specificity evaluation [28].

### 2.11. Statistical Analysis

Statistical analysis was performed using Graphpad Prism V.8.0.2 (San Diego, CA, USA). Parameters were calculated using the online Epitools platform (http://www.ausvet.com.au/ URL (accessed on 5 July 2025)).

## 3. Results

### 3.1. Development of cELISA

**Figure 1:**
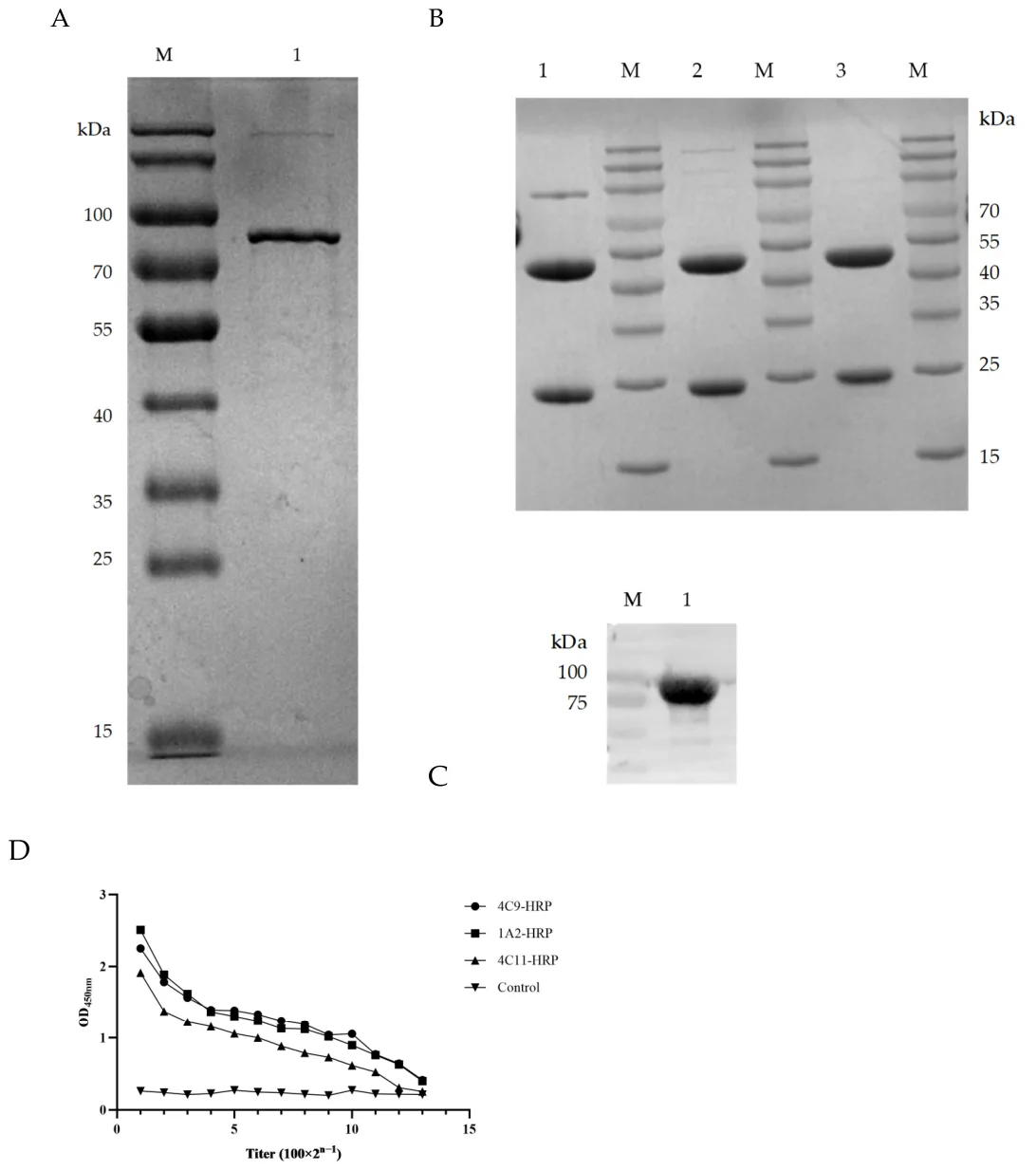
Expression, purification, immunoreactivity, and antibody titer analysis of recombinant MbovP579 and monoclonal antibodies. (**A**,**B**) SDS-PAGE analysis of purified recombinant MbovP579 protein and monoclonal antibodies. (**C**) Immunoreactivity of recombinant MbovP579 protein identified by Western blot analysis. In panels (**A**–**C**), lane M represents the molecular weight marker. In panels (**A**,**C**), lane 1 represents recombinant MbovP579 protein. In panel (**B**), lane 1 represents 4C11, lane 2 represents 4C9, and lane 3 represents 1A2. (**D**) Antibody titers of HRP-conjugated mAbs determined by iELISA. The original images of Western blot are shown in the Supplementary File.

The recombinant fusion protein MbovP579 was successfully expressed and purified, appearing as a single band at 81 kDa on a 10% SDS-PAGE with a stock concentration of 1.04 mg/mL (Figure 1A). The protein demonstrated excellent immunoreactivity in Western blot analysis against *M. bovis*-positive serum, showing a distinct band at approximately 81 kDa (Figure 1C).

The three purified mAbs (4C9, 1A2, and 4C11) showed heavy and light chain bands at 50 kDa and 25 kDa, respectively (Figure 1B). All were confirmed as the IgG1 subclass. The titers of the 4C9-HRP and 1A2-HRP monoclonal antibodies were both 1:512,000, while the titer of the 4C11-HRP monoclonal antibody was 1:204,800 (Figure 1D). The HRP-conjugated 1A2 mAb demonstrated the highest blocking rate (76.55%) among the three mAbs (Table 2), supporting its selection. Checkerboard titration established the optimal MbovP579 coating concentration at 1 μg/mL, the HRP-1A2 mAb concentration at 0.35 μg/mL (Table A1), the serum dilution at 1:4 (Table A2), and the reaction time at 60 min.

### 3.2. Determination of the Cut-Off Value

**Figure 2:**
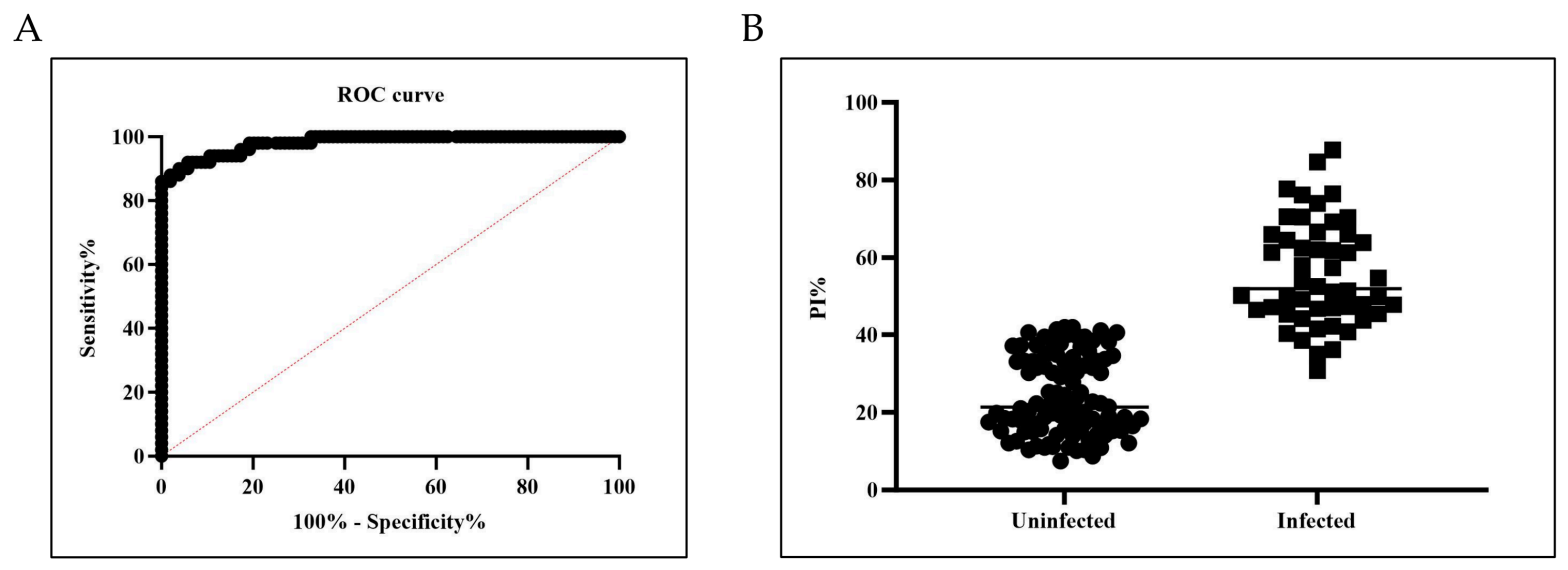
Receiver operating characteristic (ROC) analysis of the negative (uninfected) and positive (infected/immunized) sample critical values; ROC curve analysis (**A**), interactive dot diagram analysis (**B**).

A total of 154 serum samples, including 50 *M. bovis*-positive and 104 *M. bovis*-negative samples, were used to determine the cut-off value based on PI values by ROC curve analysis (Table A3). The optimal cut-off was determined to be 40.69% PI. At this threshold, the area under the ROC curve (AUC) was 0.9817 (95% CI: 0.96394–0.9996). The diagnostic sensitivity and specificity were 90% (95% CI: 78.64–95.65%) and 96.15% (95% CI: 90.53–98.49%), respectively (Figure 2A). The interactive dot diagram clearly demonstrates a distinct separation between the two groups, with the infected group exhibiting significantly higher PI% values compared to the uninfected group (Figure 2B).

### 3.3. Evaluation of cELISA

Serum samples from nine calves vaccinated with the attenuated *M. bovis* HB150 strain were tested at 0, 7, 14, 21, 28, and 35 days post-vaccination (dpv). The positive seroconversion of the calves was tested in parallel by using this cELISA and the commercial Biovet^®^ kit, and both assays showed a positivity of 100% (9/9) at 21–35 dpv (Table 3).

Serial dilution testing showed that the strongly positive serum sample remained positive up to a 32-fold dilution, whereas it tested negative at a 64-fold dilution. Thus, the terminal detection dilution of the cELISA was determined to be 1:32, which was consistent with that of the commercial indirect ELISA kit (Biovet^®^, Canada) (Table 4).

Specificity testing against related *mycoplasmas* and respiratory pathogens, *Mycoplasma bovirhinis*, *Mycoplasma agalactiae*, *Mycoplasma arginini*, *Pasteurella multocida*, and *Mannheimia haemolytica*, yielded PI values of 17.28%, 21.23%, 13.65%, 5.77%, and 24.43%, respectively. All were below the 40.69% cut-off, confirming excellent analytical specificity. Reproducibility was robust, with both intra-assay and inter-assay CVs remaining below 10% (Table A4).

### 3.4. Application for the cELISA

A total of 1069 clinical bovine serum samples were assayed (Table 5). The positive detection rate of the cELISA was 55.57% (95% CI: 52.5–58.6%), compared to 59.68% (95% CI: 56.7–62.6%) for the Biovet^®^ commercial kit. The positive agreement was 88.56% (565/638). The negative agreement was 93.27% (402/431). The overall agreement rate between the two methods was 90.46% (967/1069, 95% CI: 88.54–92.15%), with a kappa value of 0.81 (95% CI: 0.76–0.86) (Table 6), indicating high consistency. Meanwhile, 73 samples that were tested positive by the Biovet^®^ kit yet negative by cELISA were subjected to the *M. bovis* metabolic inhibition test (MIT). The MIT yielded a negative rate of 76.7%. Conversely, of the 29 samples that were tested negative by the Biovet^®^ kit but positive by cELISA, 89.7% (26/29) tested positive with the MIT. These findings suggest a tendency toward false positivity in the commercial Biovet^®^ kit.

## 4. Discussion

The diagnosis of *M. bovis* infection relies on diverse methodologies, each with distinct advantages. PCR-based techniques are widely used for pathogen detection because of their high sensitivity of 83–100% and specificity of 93–99% [29,30,31], while serological assays provide complementary value for early outbreak detection and herd surveillance. ELISA demonstrates robust performance, with reported sensitivities of 90–97% and specificities of 94–98% in field validations [10,32,33,34].

In previous work, we identified MbovP579 as a highly immunogenic membrane protein harboring multiple T-cell and B-cell epitopes [10]. Importantly, mAb 1A2, raised against MbovP579, exhibited no cross-reactivity with other *Mycoplasma* species, including *M. agalactiae* [10], underscoring its exceptional species specificity. Furthermore, the high genetic conservation of MbovP579 across *M. bovis* strains (95–100% sequence identity) supports its utility as a universal diagnostic target.

Building upon these findings, we developed a cELISA using mAb 1A2 and recombinant MbovP579. By conjugating mAb 1A2 directly with HRP, we eliminated the need for secondary antibodies, streamlining the procedure. The assay performed comparably to a commercial kit, achieving a 100% seropositivity rate by day 21 post-vaccination and maintaining detection at a 1:32 dilution.

In the clinical validation using 1069 bovine serum samples, the cELISA showed high overall agreement with the commercial kit (90.46%; kappa = 0.81). Discrepant samples were retested using the MIT assay, whose results confirmed that the commercially available Biovet^®^ kit yielded more false-positive results, further corroborating the superior specificity of the newly developed cELISA.

Compared with conventional indirect ELISAs, the MbovP579-based cELISA offers enhanced specificity without compromising sensitivity, aligning with emerging trends in veterinary serology that favor recombinant protein-based assays for their reproducibility and reduced batch-to-batch variability. This cELISA is currently being validated using clinical specimens; pending favorable validation results, the reagent kit will proceed to regulatory submission for commercialization—thereby improving diagnostic accuracy and supporting disease prevention and control efforts.

## 5. Conclusions

The MbovP579-based cELISA developed in this study represents a significant advancement in serological diagnosis of *M. bovis* infection, offering a reliable and affordable tool for combating *M. bovis* infections. The developed assay demonstrates apparent sensitivity, specificity, and reproducibility, providing promising applications for clinical diagnostics, large-scale surveillance, and supporting sustainable cattle production.

## Figures and Tables

**Table 1:** Information on positive serum samples used in this study.

Test Group	No. of Samples	Pathogenic Species	Criteria for Positivity
Determination **of the cut-off value**	50	*M. bovis* HB150 [11,21]	Antiserum from the calves immunized with attenuated *M. bovis* and determined as positive with the Biovet^®^ kit ^1^.
**Evaluation of the sensitivity of cELISA**	1	*M. bovis* HB150 [11,21]	Antiserum from the calves immunized with attenuated *M. bovis* and determined as positive with the Biovet^®^ kit.
**Evaluation of the specificity of the cELISA**	1	*Mycoplasma agalactiae* (CVCC344) ^2^	Antiserum from the calves immunized with inactivated *Mycoplasma agalactiae* was determined as positive with in-house iELISA.
	1	*Mycoplasma bovirhinis* (CVCC361) ^3^	Antiserum from the calves immunized with inactivated *Mycoplasma bovirhinis* and determined as positive with in-house iELISA.
	1	*Mycoplasma arginini* (CVCC346) ^4^	Antiserum from the calves immunized with inactivated *Mycoplasma arginini* was determined as positive with in-house iELISA.
	1	*Pasteurella multocida* (CVCC390) ^5^	Antiserum from the calves immunized with inactivated *Pasteurella multocida* was determined as positive with in-house iELISA.
	1	*Mannheimia haemolytica* ^6^	Antiserum from the calves immunized with inactivated *Mannheimia haemolytica* and determined as positive with in-house iELISA.

^1^: *Bovine Mycoplasma* indirect ELISA antibody test kit (Biovet^®^, QC, Canada). ^2,3,4,5^: Bacterial strains used in this study were purchased from the National Center for Veterinary Culture Collection (CVCC) and maintained by the National Key Laboratory of Agricultural Microbiology, Huazhong Agricultural University. ^6^: The *M. haemolytica* strain was isolated and maintained by the National Key Laboratory of Agricultural Microbiology, Huazhong Agricultural University.

**Table 2:** Evaluation of the competition effect of monoclonal antibodies.

	mAbs								
Dilutions	4C9			1A2			4C11		
	P	N	PI Value	P	N	PI Value	P	N	PI Value
1000	1.24	1.57	21.42%	0.63	1.85	66.02%	1.34	1.61	17.07%
2000	0.84	1.47	42.40%	0.39	1.37	71.40%	1.21	1.50	19.06%
3000	0.92	1.38	33.13%	0.32	1.26	74.97%	0.97	1.25	22.19%
4000	0.65	1.28	49.07%	0.18	0.79	76.55%	0.74	1.19	37.81%
5000	0.48	1.03	53.05%	0.12	0.37	67.44%	0.53	0.83	36.55%
6000	0.29	0.88	66.94%	0.08	0.21	61.92%	0.32	0.59	46.34%
8000	0.18	0.63	71.24%	0.07	0.13	50.30%	0.20	0.38	47.66%
16,000	0.13	0.41	67.38%	0.06	0.10	41.32%	0.12	0.25	50.12%

Note: P represents positive, while N represents negative.

**Table 3:** Results of the cELISA sensitivity test.

Test Method	Time After Immunization (Days)					
	0	7	14	21	28	35
cELISA	0/9	4/9	7/9	9/9	9/9	9/9
Biovet^®^ kit	0/9	4/9	8/9	9/9	9/9	9/9

**Table 4:** Analytical sensitivity comparison of the cELISA and Biovet^®^ kit sensitivity tests.

Dilutions		1	2	4	8	16	32	64	128
Relevance ratio	cELISA	P	P	P	P	P	P	N	N
	Biovet^®^ kit	P	P	P	P	P	P	N	N

Note: “P” represents positive, “N” represents negative.

**Table 5:** Positive rates of serum samples detected by two methods.

Province	Farms	Serum Samples	cELISA	Biovet^®^ Kit
			Positive Rate (%)	Positive Rate (%)
Hubei	SZ	160	19.38 (95% CI: 13.6–26.4)	22.50 (95% CI: 16.3–29.8)
Jiangxi	GA	304	50.99 (95% CI: 45.2–56.7)	56.25 (95% CI: 50.5–61.9)
Inner Mongolia	JY	350	56.57 (95% CI: 51.2–61.8)	60.00 (95% CI: 54.7–65.2)
Hubei	QNE	255	82.35 (95% CI: 77.1–86.8)	86.67 (95% CI: 81.9–90.6)
Total		1069	55.57 (95% CI: 52.5–58.6)	59.68 (95% CI: 56.7–62.6)

**Table 6:** Comparison of the results of *M. bovis* antibody detection by cELISA and the Biovet^®^ kit.

Serum Samples		Biovet^®^ Kit			
		+	-	Total	Agreement
MbovP579-cELISA	+	565	29	594	90.46% (95% CI: 88.54–92.15%)
	-	73	402	475	
	Total	638	431	1069	
Kappa (95% CI)	0.81 (95% CI: 0.76–0.86)				

Note: “+” represents positive; “-” represents negative.

## Data Availability

The original contributions presented in this study are included in the article/Supplementary Material. Further inquiries can be directed to the corresponding author.

## Supplementary Materials

The following supporting information can be downloaded at: https://www.mdpi.com/article/10.3390/vetsci13080741/s1, Figure S1: Expression, purification, and immunoreactivity of recombinant MbovP579 and monoclonal antibodies.

## Appendix A

**Table A1:** Determination of MbovP579 and HRP-1A2 dilutions.

Concentration of HRP-1A2 (μg/mL)	Concentrations of MbovP579 (μg/mL)						
		4.0	2.0	1.3	1.0	0.67	0.5
2.8	N	2.389	2.546	2.032	1.977	1.763	1.654
	P	0.764	0.643	0.613	0.467	0.454	0.337
	N/P	3.127	3.960	3.315	4.233	3.883	4.908
1.4	N	2.342	2.187	1.932	1.877	1.621	1.442
	P	0.786	0.678	0.579	0.451	0.412	0.321
	N/P	2.980	3.226	3.337	4.162	3.934	4.492
0.7	N	2.079	1.896	1.885	1.732	1.537	1.211
	P	0.646	0.542	0.531	0.344	0.328	0.217
	N/P	3.218	3.498	3.550	5.035	4.686	5.581
0.5	N	1.568	1.526	1.448	1.413	1.229	1.137
	P	0.587	0.477	0.425	0.279	0.226	0.195
	N/P	2.671	3.199	3.407	5.065	5.438	5.831
0.4	N	1.443	1.321	1.279	1.288	1.027	0.998
	P	0.532	0.413	0.404	0.203	0.179	0.105
	N/P	2.712	3.199	3.166	6.345	5.737	9.505
0.35	N	1.278	1.228	1.134	1.275	0.975	0.866
	P	0.441	0.398	0.331	0.108	0.087	0.076
	N/P	2.898	3.085	3.426	11.806	11.207	11.395
0.31	N	1.132	1.036	0.987	0.832	0.766	0.678
	P	0.322	0.321	0.295	0.079	0.069	0.069
	N/P	3.516	3.227	3.346	10.532	11.101	9.826
0.28	N	0.923	0.823	0.745	0.523	0.489	0.478
	P	0.279	0.228	0.204	0.065	0.054	0.043
	N/P	3.308	3.610	3.652	8.046	9.056	11.116

Note: “P”: the values of OD of the positive control serum; “N”: the values of OD of the negative control serum.

**Table A2:** Determination of serum dilutions.

Group	Dilutions			
	2	4	8	10
P	0.316	0.112	0.107	0.073
N	1.237	1.187	1.077	0.765
N/P	3.915	10.598	10.065	3.542

Note: “P”: the values of OD of the positive control serum; “N”: the values of OD of the negative control serum.

**Table A3:** Serum samples for the determination of the cut-off value.

Positive			Negative					
Number	OD_450_	PI Value	Number	OD_450_	PI Value	Number	OD_450_	PI Value
M1	0.4113	51.20%	N1	0.5919	36.90%	N53	0.6545	30.23%
M2	0.3257	61.36%	N2	0.5497	41.40%	N54	0.7012	25.25%
M3	0.4257	49.50%	N3	0.6069	35.30%	N55	0.8199	12.59%
M4	0.4873	42.19%	N4	0.6106	34.90%	N56	0.7702	17.89%
M5	0.3214	61.87%	N5	0.545	41.90%	N57	0.7662	18.32%
M6	0.2592	69.25%	N6	0.545	41.90%	N58	0.7906	15.71%
M7	0.1293	84.67%	N7	0.5619	40.10%	N59	0.7285	22.33%
M8	0.419	50.30%	N8	0.5525	41.10%	N60	0.7299	22.19%
M9	0.1985	76.46%	N9	0.5572	40.60%	N61	0.8278	11.75%
M10	0.3996	52.60%	N10	0.5779	38.39%	N62	0.8407	10.37%
M11	0.4993	40.77%	N11	0.5575	40.57%	N63	0.7442	20.66%
M12	0.4399	47.82%	N12	0.5753	38.66%	N64	0.8352	10.96%
M13	0.4611	45.30%	N13	0.653	30.39%	N65	0.7523	19.80%
M14	0.4476	46.90%	N14	0.6275	33.10%	N66	0.8247	12.08%
M15	0.451	46.50%	N15	0.5881	37.30%	N67	0.8048	14.20%
M16	0.326	61.33%	N16	0.5681	39.44%	N68	0.8409	10.36%
M17	0.2988	64.55%	N17	0.6262	33.25%	N69	0.7758	17.30%
M18	0.3589	57.43%	N18	0.6548	30.19%	N70	0.7021	25.15%
M19	0.2866	66.00%	N19	0.6501	30.69%	N71	0.7147	23.81%
M20	0.1878	77.72%	N20	0.6218	33.71%	N72	0.7953	15.22%
M21	0.249	70.47%	N21	0.5834	37.80%	N73	0.7776	17.10%
M22	0.4704	44.20%	N22	0.5675	39.50%	N74	0.7969	15.05%
M23	0.4445	47.27%	N23	0.6172	34.20%	N75	0.7689	18.03%
M24	0.538	36.18%	N24	0.5797	38.20%	N76	0.8245	12.11%
M25	0.5032	40.31%	N25	0.5892	37.19%	N77	0.8429	10.14%
M26	0.1032	87.76%	N26	0.5652	39.75%	N78	0.7902	15.76%
M27	0.3164	62.46%	N27	0.6262	33.25%	N79	0.8051	14.17%
M28	0.4386	47.97%	N28	0.7035	25.00%	N80	0.7408	21.02%
M29	0.4739	43.78%	N29	0.677	27.83%	N81	0.7593	19.06%
M30	0.2007	76.19%	N30	0.7323	21.93%	N82	0.832	11.30%
M31	0.4599	45.44%	N31	0.6547	30.21%	N83	0.7494	20.10%
M32	0.5482	34.97%	N32	0.5889	37.22%	N84	0.762	18.76%
M33	0.248	70.58%	N33	0.6398	31.79%	N85	0.8063	14.04%
M34	0.2862	66.05%	N34	0.6025	35.76%	N86	0.8348	11.00%
M35	0.4451	47.21%	N35	0.6268	33.18%	N87	0.7648	18.46%
M36	0.4224	49.90%	N36	0.7383	21.29%	N88	0.795	15.25%
M37	0.4485	46.80%	N37	0.7288	22.31%	N89	0.833	11.19%
M38	0.4282	49.21%	N38	0.6366	32.13%	N90	0.79	15.78%
M39	0.3045	63.88%	N39	0.7537	19.65%	N91	0.7574	19.25%
M40	0.3541	57.99%	N40	0.7246	22.75%	N92	0.7622	18.74%
M41	0.2812	66.64%	N41	0.6285	32.99%	N93	0.8522	7.47%
M42	0.3898	53.76%	N42	0.7083	24.49%	N94	0.7711	16.27%
M43	0.5837	30.76%	N43	0.7975	14.98%	N95	0.7529	18.25%
M44	0.5181	38.54%	N44	0.7474	20.32%	N96	0.7926	13.94%
M45	0.3198	62.06%	N45	0.7367	21.46%	N97	0.8405	8.74%
M46	0.2499	70.36%	N46	0.7735	17.54%	N98	0.8017	12.95%
M47	0.4092	51.46%	N47	0.6421	31.55%	N99	0.7743	15.93%
M48	0.493	41.51%	N48	0.6262	33.24%	N100	0.8204	10.93%
M49	0.2194	73.97%	N49	0.7522	19.81%	N101	0.7522	18.32%
M50	0.381	54.80%	N50	0.6647	29.14%	N102	0.7801	15.30%
			N51	0.6423	31.52%	N103	0.7688	16.53%
			N52	0.613	34.65%	N104	0.7641	17.03%

M1–M50: *M. bovis*-positive serum samples were collected from the calves immunized with *M. bovis* HB150. N1–N104: *M. bovis*-negative serum samples were collected from unvaccinated *M. bovis* farms and stored at −20 °C in the HZAU laboratory.

**Table A4:** Results of intra and inter repeatability tests by cELISA (*n* = 20).

Serum Number	Intra Batch (PI%)		Inter Batch (PI%)	
	$\bar{X}$ ± SD	CV (%)	$\bar{X}$ ± SD	CV (%)
N1	16.56 ± 0.51	3.03	16.99 ± 0.68	4.00
N2	26.04 ± 0.37	1.40	26.28 ± 0.51	1.94
N3	10.71 ± 0.07	0.66	10.79 ± 0.11	0.98
N4	10.45 ± 0.37	3.56	10.65 ± 0.80	7.47
N5	11.21 ± 0.38	3.38	11.46 ± 0.44	3.80
N6	7.93 ± 0.29	3.59	8.52 ± 0.46	5.37
N7	11.44 ± 0.63	5.60	11.77 ± 0.37	3.12
N8	25.60 ± 0.35	1.37	26.04 ± 0.09	0.35
N9	5.12 ± 0.36	6.94	4.53 ± 0.42	9.18
N10	8.48 ± 0.67	7.60	10.27 ± 0.65	6.29
P1	84.57 ± 0.24	0.28	81.97 ± 0.44	0.53
P2	81.96 ± 0.79	0.95	83.42 ± 0.42	0.51
P3	67.72 ± 1.06	1.55	67.73 ± 0.35	0.51
P4	57.02 ± 1.05	1.86	53.49 ± 0.97	1.82
P5	53.05 ± 0.56	1.05	54.62 ± 0.37	0.68
P6	41.65 ± 0.32	0.76	38.28 ± 1.95	5.10
P7	47.68 ± 1.62	3.46	50.01 ± 1.58	3.17
P8	43.84 ± 1.45	3.26	46.70 ± 0.34	0.72
P9	42.83 ± 0.39	0.90	39.13 ± 1.61	4.11
P10	48.28 ± 1.91	4.04	42.29 ± 2.07	4.89

Note: P1–P10 represent 10 positive samples; N1–N10 represent 10 negative samples.

## References

[1] Maunsell F.P., Woolums A.R., Francoz D., Rosenbusch R.F., Step D.L., Wilson D.J., Janzen E.D. (2011). *Mycoplasma bovis* infections in cattle. J. Vet. Intern. Med..

[2] Castillo Alcala F., Bateman K.G., Cai H.Y., Schott C.R., Parker L., Clark M.E., McRaild P., McDowall R.M., Foster R.A., Archambault M. (2012). Prevalence and genotype of *Mycoplasma bovis* in beef cattle after arrival at a feedlot. Am. J. Vet. Res..

[3] Lysnyansky I., Ayling R.D. (2016). *Mycoplasma bovis*: Mechanisms of resistance and trends in antimicrobial susceptibility. Front. Microbiol..

[4] Mulongo M., Prysliak T., Perez Casal J. (2013). Vaccination of feedlot cattle with extracts and membrane fractions from two *Mycoplasma bovis* isolates results in strong humoral immune responses but does not protect against an experimental challenge. Vaccine.

[5] Gelgie A.E., Korsa M.G., Kerro Dego O. (2022). *Mycoplasma bovis* mastitis. Curr. Res. Microb. Sci..

[6] Pothmann H., Spergser J., Elmer J., Prunner I., Iwersen M., Klein Jöbstl D., Drillich M. (2015). Severe *Mycoplasma bovis* outbreak in an Austrian dairy herd. J. Vet. Diagn. Investig..

[7] Torres F.D., Madureira K.M., Nascimento da Silva K., Azevedo C., de Moura T.M., Skorei M.R., Gomes V. (2022). Influence of *Mycoplasma bovis* infection on milk production and quality of holstein dairy cows. J. Dairy Res..

[8] Bokma J., Stuyvaert S., Pardon B. (2022). Comparison and optimisation of screening cut-off values for *Mycoplasma bovis* antibody ELISAs using serum from youngstock. Vet. Rec..

[9] Haines D.M., Moline K.M., Sargent R.A., Campbell J.R., Myers D.J., Doig P.A. (2004). Immunohistochemical study of *Hemophilus somnus*, *Mycoplasma bovis*, *Mannheimia hemolytica*, and *Bovine viral diarrhea virus* in death losses due to myocarditis in feedlot cattle. Can. Vet. J..

[10] Khan F.A., Faisal M., Chao J., Liu K., Chen X., Zhao G., Menghwar H., Zhang H., Zhu X., Rasheed M.A. (2016). Immunoproteomic identification of MbovP579, a promising diagnostic biomarker for serological detection of *Mycoplasma bovis* infection. Oncotarget.

[11] Qi J., Guo A., Cui P., Chen Y., Mustafa R., Ba X., Hu C., Bai Z., Chen X., Shi L. (2012). Comparative geno-plasticity analysis of *Mycoplasma bovis* HB0801 (Chinese Isolate). PLoS ONE.

[12] Valeris Chacin R., Powledge S., McAtee T., Morley P.S., Richeson J. (2022). *Mycoplasma bovis* is associated with *Mannheimia haemolytica* during acute bovine respiratory disease in feedlot cattle. Front. Microbiol..

[13] Perez Casal J., Prysliak T., Maina T., Suleman M., Jimbo S. (2017). Status of the development of a vaccine against *Mycoplasma bovis*. Vaccine.

[14] Parker A.M., Sheehy P.A., Hazelton M.S., Bosward K.L., House J.K. (2018). A Review of *Mycoplasma* diagnostics in cattle. Vet. Intern. Med..

[15] Dudek K., Nicholas R.A.J., Szacawa E., Bednarek D. (2020). *Mycoplasma bovis* infections-occurrence, diagnosis and control. Pathogens.

[16] Nicholas R.A.J., Ayling R.D. (2003). *Mycoplasma bovis*: Disease, diagnosis, and control. Res. Vet. Sci..

[17] Calcutt M.J., Lysnyansky I., Sachse K., Fox L.K., Nicholas R.A.J., Ayling R.D. (2018). Gap Analysis of *Mycoplasma bovis* disease, diagnosis and control: An aid to identify future development requirements. Transbound. Emerg. Dis..

[18] Le G.D., Calavas D., Brank M., Citti C., Rosengarten R., Bézille P., Poumarat F. (2002). Serological prevalence of *Mycoplasma bovis* infection in suckling beef cattle in France. Vet. Rec..

[19] Burns R. (2005). Immunochemical Protocols.

[20] Zhang W. (2019). Preparation of Monoclonal Antibodies Against the P579 Protein of *Mycoplasma bovis* and Preliminary Establishment of a Sandwich ELISA. Master’s Thesis.

[21] Zhang R., Han X., Chen Y., Mustafa R., Qi J., Chen X., Hu C., Chen H., Guo A. (2014). Attenuated *Mycoplasma bovis* strains provide protection against virulent infection in calves. Vaccine.

[22] García Galán A., Seva J., Gómez Martín Á., Ortega J., Rodríguez F., García Muñoz Á., De la Fe C. (2021). Importance and antimicrobial resistance of *Mycoplasma bovis* in clinical respiratory disease in feedlot calves. Animals.

[23] Gelgie A.E., Desai S.E., Gelalcha B.D., Kerro Dego O. (2024). *Mycoplasma bovis* mastitis in dairy cattle. Front. Vet. Sci..

[24] Shkap V., Cohen I., Leibovitz B., Pipano E., Avni G., Shofer S., Giger U., Kappmeyer L., Knowles D. (1998). Seroprevalence of *Babesia equi* among horses in Israel using competitive inhibition ELISA and IFA assays. Vet. Parasitol..

[25] Afshar A., Thomas F.C., Wright P.F., Shapiro J.L., Shettigara P.T., Anderson J. (1987). Comparison of competitive and indirect enzyme-linked immunosorbent assays for detection of *Bluetongue virus* antibodies in serum and whole blood. J. Clin. Microbiol..

[26] Glaros A.G., Kline R.B. (1988). Understanding the accuracy of tests with cutting scores: The sensitivity, specificity, and predictive value model. J. Clin. Psychol..

[27] Mustafa R.R., Sukor R., Mohd Nor S.M., Saari N., Mohsin A.Z. (2023). Development of methyl ester antibody-based competitive indirect ELISA for quantitative detection of *Mitragynine* in human urine. ACS Omega.

[28] Miles R., Nicholas R. (1998). Mycoplasma Protocols Methods in Molecular Biology^TM^.

[29] Song S., Guo J., Zhao Y., Shi F., Wang Y., Zhang Q., Wang Z., Chen C. (2024). Development of polymerase chain reaction-lateral flow dipstick assay for detection of *Mycoplasma bovis* in cattle. BMC Vet. Res..

[30] Clothier K.A., Jordan D.M., Thompson C.J., Kinyon J.M., Frana T.S., Strait E.L. (2010). *Mycoplasma bovis* real-time polymerase chain reaction assay validation and diagnostic performance. J. VET. Diagn. Invest..

[31] Chauhan K., Aly S.S., Lehenbauer T.W., Tonooka K.H., Glenn K., Rossitto P., Marco M.L. (2021). Development of a multiplex qPCR assay for the simultaneous detection of *Mycoplasma bovis, Mycoplasma* species, and *Acholeplasma laidlawii* in Milk. PeerJ.

[32] Wawegama N.K., Browning G.F., Kanci A., Marenda M.S., Markham P.F. (2014). Development of a recombinant protein-based enzyme-linked immunosorbent assay for diagnosis of *Mycoplasma bovis* infection in cattle. Clin. Vaccin. Immunol..

[33] Andersson A.M., Aspán A., Wisselink H.J., Smid B., Ridley A., Pelkonen S., Autio T., Lauritsen K.T., Kensø J., Gaurivaud P. (2019). A european inter-laboratory trial to evaluate the performance of three serological methods for diagnosis of *Mycoplasma bovis* infection in cattle using latent class analysis. BMC Vet. Res..

[34] Salgadu A., Firestone S.M., Watt A., Thilakarathne D.S., Condello A.K., Siu D., Masukagami Y., Tivendale K.A., Stevenson M.A., Mansell P.D. (2022). Evaluation of the MilA ELISA for the diagnosis of herd infection with *Mycoplasma bovis* using bulk tank milk and estimation of the prevalence of *M. bovis* in Australia. Vet. Microbiol..

