# Supplementary material for: Serological Detection of Antibodies Against Mycoplasma bovis Infection by Competitive Enzyme-Linked Immunosorbent Assay (cELISA)
Source: Vet Sci. 2026 Jul 25;13(8):741. doi: 10.3390/vetsci13080741 (PMC13517677; doi:10.3390/vetsci13080741)

Supplementary S1: Expression, purification, and immunoreactivity of recombinant MbovP579 and monoclonal antibodies.

A. SDS-PAGE analysis of purified recombinant MbovP579

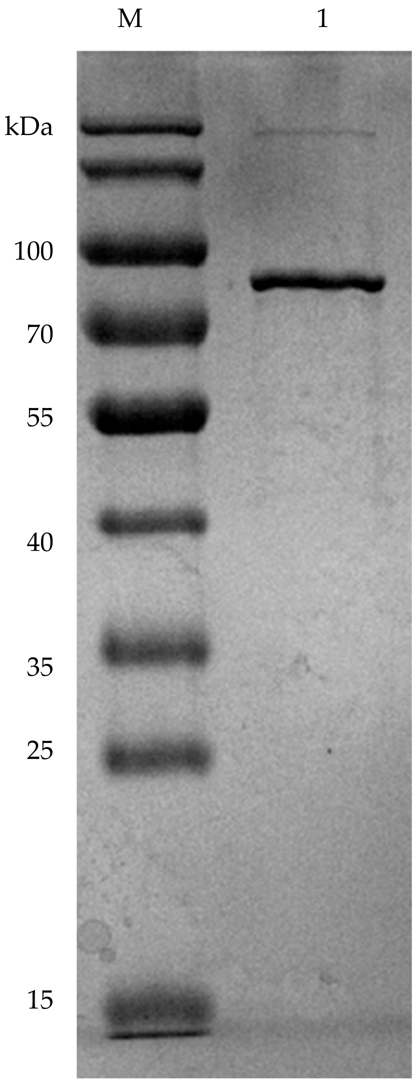

B. SDS-PAGE analysis of purified recombinant MbovP579 monoclonal antibodies.

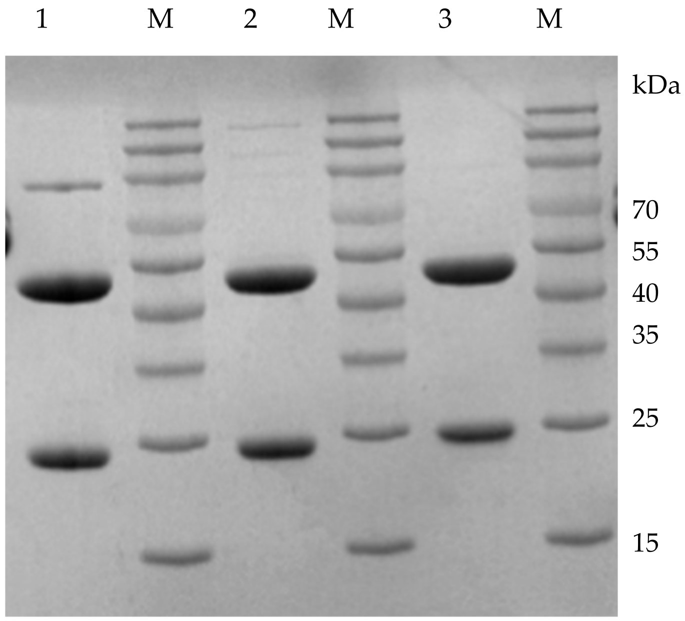

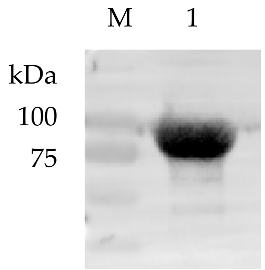

C. Immunoreactivity of recombinant MbovP579 protein identified by Western blot analysis.

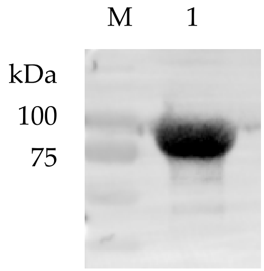

Supplement: Supplementary file 1 [file vetsci-13-00741-s001.zip › vetsci-4446177-supplementary.pdf]
